# Neutrophils and Neutrophil Extracellular Traps in Cancer and Cardio-Oncology: Molecular Mechanisms, Therapeutic Targeting, and Clinical Perspectives

**DOI:** 10.3390/medsci14060628

**Published:** 2026-10-01

**Authors:** Melpomeni Maria Panou, Kallirroi Kalantzi, Despoina Pantazi

**Affiliations:** 1 Laboratory of Biochemistry, Department of Chemistry, University of Ioannina, 45110 Ioannina, Greece; panoumelina@gmail.com; 2 Department of Nursing, School of Health Sciences, University of Ioannina, 45110 Ioannina, Greece; kalkalantzi@uoi.gr

**Keywords:** cancer, cardio-oncology, mechanisms, neutrophils, neutrophil extracellular traps

## Abstract

Neutrophils are key regulators of innate immunity and, in addition to their antimicrobial function, contribute to the progression of tumors by forming extracellular traps (NETs). Within the tumor microenvironment, the NETs interact with tumor cells, immune cells, fibroblasts and endothelial cells to promote tumor growth, angiogenesis, immune suppression, metastasis and resistance to treatment. This review critically examines the molecular mechanisms underlying intercellular communication mediated by NETs in cancer and examines their impact on disease progression and response to treatment. In addition, we assess current therapeutic approaches focused on NETs, discuss the available clinical evidence and highlight the main limitations, including safety concerns, validation of biomarkers and translational challenges. We also outline unresolved issues and future research priorities, emphasizing the need for more mechanistic and clinical studies. In addition to oncology, NETs can also play a role in cardio-oncology by linking cancer-related inflammation to thrombosis and cardiotoxicity. Overall, this review provides an integrated framework for understanding the biological and clinical relevance of NETs and supports their potential as a therapeutic target and biomarker in precision medicine.

## 1. Introduction

Neutrophils are the most abundant circulating leukocytes and play central roles in innate immunity, inflammatory responses, tissue remodeling, and immune regulation [1,2,3]. Beyond their classical roles in pathogen recognition, phagocytosis, and degranulation, neutrophils interact closely with tumor cells and stromal elements within the tumor microenvironment (TME). Through cross-talk with macrophages, fibroblasts, lymphocytes, endothelial cells, and platelets, neutrophils can modulate inflammatory signaling, immune suppression, tumor progression, and therapeutic responses [2,4,5,6].

Neutrophil extracellular traps (NETs) are web-like extracellular structures composed primarily of decondensed DNA complexed with granular and cytoplasmic proteins, histones, myeloperoxidase, and neutrophil elastase. Initially identified in 2004 as an antimicrobial mechanism that immobilizes and eradicates pathogens [7,8,9]. NET release can be triggered by both sterile and infectious stimuli, including granulocyte colony-stimulating factor (G-CSF), inflammatory cytokines, tumor-derived factors, tissue damage, and platelet activation. Depending on the specific stimulus and cellular context, NET formation involves PAD4-mediated chromatin decondensation, ROS-dependent or ROS-independent signaling, and lytic or non-lytic forms of extracellular DNA release [10].

Although NETs facilitate host defense, excessive or persistent NET formation can promote thrombosis, endothelial damage, pathological inflammation, and adverse tissue remodeling. In cancer, NETs influence the tumor microenvironment (TME), tumor-cell survival, invasion, metastatic dissemination, angiogenesis, immune evasion, and resistance to anticancer therapy through multi-cellular interactions [11,12,13,14,15]. Crucially, the biological impact of NETs varies depending on tumor type, disease stage, anatomical location, the inflammatory milieu, and treatment exposure.

The significance of NETs is particularly pronounced in cardio-oncology, where malignancy and anticancer treatments elicit overlapping inflammatory, thromboinflammatory, and vascular stress responses. Dysregulated NET formation has been linked to endothelial dysfunction, platelet activation, cancer-associated thrombosis (CAT), microvascular damage, myocardial inflammation, fibrosis, and treatment-related cardiotoxicity [16,17,18,19,20]. While the exact extent to which NETs directly mediate therapy-induced cardiovascular injury remains to be fully elucidated, these pathways can be further altered by anthracyclines, radiation, immune checkpoint inhibitors, and other systemic treatments. Consequently, NETs may serve as potential biomarkers and therapeutic targets while acting as a critical mechanistic link between tumor progression, thromboembolic complications, and cardiovascular toxicity [21].

This review explores the complementary roles of neutrophils and neutrophil extracellular traps (NETs) within the fields of oncology and cardio-oncology. To provide a thorough overview of how general neutrophil function drives tumor progression, metastasis, therapeutic resistance, thrombosis, and cancer-associated cardiovascular complications, a detailed analysis of these cellular behaviors is performed alongside specific NET-dependent pathways.

## 2. Neutrophils and Neutrophil Extracellular Traps

Neutrophils are the cellular source of these traps; therefore, interpreting the systemic effects of NETs requires a comprehensive understanding of their recruitment, activation, functional diversity, and interactions within the tumor microenvironment. To this end, this section first describes relevant neutrophil functions before focusing on the formation, structure, and pathological effects of NETs.

### 2.1. Neutrophils as Cellular Precursors of NETs

Neutrophils are the most common leukocytes in the circulation, comprising about 50–70% of all white blood cells. These short-lived cells are produced in the bone marrow and have a half-life in the blood of approximately 12 h. Neutrophils play a key role in the innate immune response by providing the first line of defense against pathogens through mechanisms such as phagocytosis, degranulation, and the release of antimicrobial agents [22,23]. Neutrophils are derived from hematopoietic stem cells in the bone marrow and undergo a well-controlled maturation process [24]. Their differentiation leads to functional diversity, allowing them to respond effectively to infection and inflammatory stimuli [25]. Under normal conditions, the production of neutrophils is tightly controlled. However, in pathological conditions such as cancer or infection, emergency granulopoiesis may result in the release of immature neutrophils into the bloodstream [17,26,27].

Upon recruitment into tumors, neutrophils may have both pro- and anti-tumorigenic properties [28]. Tumor-associated neutrophils (TANs) interact with various immune and stromal cells and influence tumor progression by releasing cytokines, proteases, and reactive oxygen species (ROS) [29,30,31]. Their functional plasticity depends on signals from the tumor microenvironment and can be polarized towards tumor-promoting (N2) or tumor-suppressing (N1) phenotypes [32,33].

Neutrophils play a key role in cancer progression by modulating angiogenesis, metastasis, and immunodeficiency [34]. Their interaction with other immune cells, including macrophages and T cells, can inhibit the immune response to cancer [13,35,36]. Additionally, the production of tumor-promoting factors is linked to an increase in tumor cell proliferation and resistance to treatment, making neutrophils a potential target for new cancer therapies [37,38,39].

### 2.2. Molecular Mechanisms, Composition, and Functions of NETs

Neutrophil extracellular traps (NETs) are webs of structures composed of DNA, histones, and antimicrobial proteins released by neutrophils to bind and neutralize pathogens [40]. The formation of NETs, known as NETosis, is triggered by a variety of factors, including infections, inflammatory signals, and tumor-derived factors [41,42,43].

Although NETs play a protective role in immunity, overproduction or dysregulation of NETs may contribute to tissue damage and chronic inflammation [44,45]. NETs have both beneficial and detrimental effects on the body [46]. They increase antimicrobial resistance by capturing and killing pathogens, but uncontrolled formation of these structures is involved in a number of diseases, including autoimmune diseases, thrombosis, and chronic inflammatory diseases [47]. Their role in promoting inflammation and immune suppression makes them particularly relevant to cancer progression [13,48].

Recent evidence suggests that the production of pro-inflammatory and immunosuppressive microenvironments by NETs facilitates tumor growth and metastasis. NETs may enhance the adhesion of tumor cells, protect tumor cells against immune attacks, and stimulate angiogenesis [13,17,49]. Understanding the mechanisms of NETs’ involvement in cancer provides the basis for developing targeted therapies that modulate the production or function of NETs [30,35,50].

### 2.3. Neutrophils and Cancer

Neutrophils, a key component of the innate immune system, play a vital role in the development of tumors, both in the body and in the environment. While their primary function is to protect against pathogens through phagocytosis and inflammatory responses, they can also contribute to the development, progression, and metastasis of tumors by a variety of mechanisms [22,51].

#### 2.3.1. Tumor-Associated Neutrophil Heterogeneity in the Tumor Microenvironment

**Figure 1:**
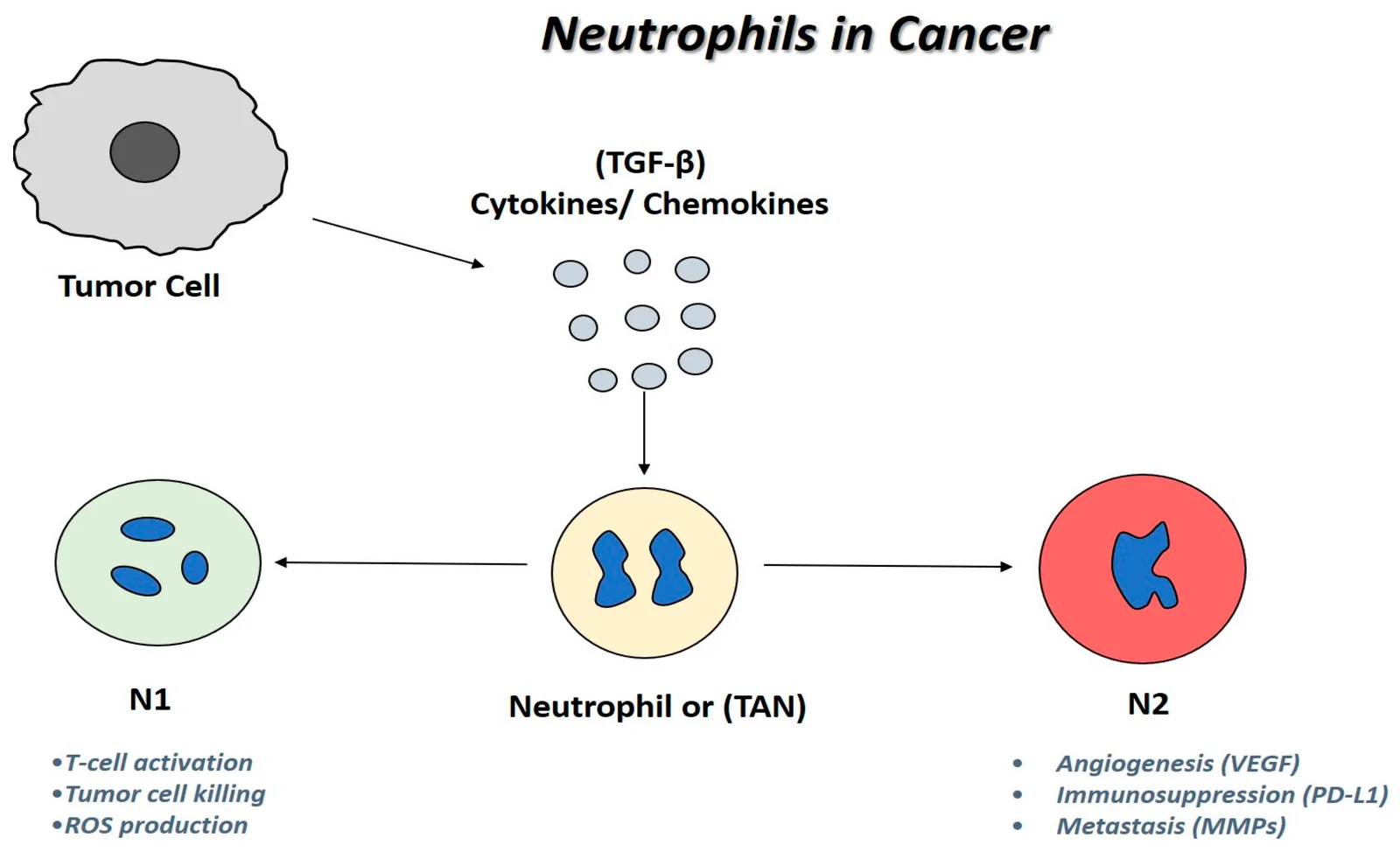
Neutrophil heterogeneity within the tumor microenvironment. Tumor-derived chemokines and cytokines recruit neutrophils to the tumor microenvironment, where they acquire diverse and plastic tumor-associated neutrophil (TAN) states. Rather than representing two discrete neutrophil populations, the N1/N2 paradigm is presented as a conceptual framework describing antitumor and protumor functional phenotypes. These states are shaped by TGF-β, hypoxia, inflammatory signals, and other microenvironmental factors that influence angiogenesis, metastasis, immune regulation, and tumor progression.

Cytokines and chemokines, such as G-CSF, IL-8, and TGF-β, recruit neutrophils to the tumor microenvironment, where they adopt highly adaptable tumor-associated phenotypes [27,52]. Although the traditional N1/N2 model describes distinct antitumor and protumor polarizations, current research suggests that tumor-associated neutrophils (TANs) exist along a dynamic continuum shaped by tumor type [32], disease stage, hypoxia, inflammation, metabolism, and therapeutic interventions [33,39]. Recent single-cell and spatial analyses have revealed several TAN states that are inadequately captured by this binary framework. Consequently, the terms “N1-like” and “N2-like” should be used with caution, particularly when linking TAN heterogeneity to NET formation [53,54,55] (Figure 1).

#### 2.3.2. Neutrophil-Associated Mechanisms in Metastasis

Neutrophils can modulate multiple stages of the metastatic cascade via both NET-independent and NET-associated mechanisms. NET-independent effects encompass the release of proteolytic enzymes, such as matrix metalloproteinases, the modulation of immune and stromal cell interactions with tumor cells, and the regulation of inflammatory and angiogenic signaling pathways [17]. These cellular processes facilitate localized tissue remodeling, promote tumor cell survival, and prepare distant tissues for metastatic colonization [13,49]. Conversely, Section 3.2 delineates the specific contribution of NETs to the circulatory trapping of tumor cells, endothelial adhesion, pre-metastatic niche formation, and extracellular matrix remodeling.

#### 2.3.3. Context-Dependent Pro-Tumor and Anti-Tumor Neutrophil Functions

Emerging evidence suggests that neutrophils exert context-dependent effects in cancer, ranging from the promotion of tumor growth and metastasis to the enhancement of antitumor immunity [54,56]. Antitumor activities include the generation of reactive oxygen and nitrogen species, Fc receptor-mediated antibody-dependent cellular cytotoxicity, and functional interactions with adaptive immune cells [57,58,59,60]. Conversely, protumor-associated functions encompass immunosuppression, cytokine-mediated tumor-cell support, angiogenesis, tissue remodeling, and therapeutic resistance. Because these diverse effects can operate via either NET-dependent or NET-independent pathways, the N1/N2 terminology alone should not be utilized to classify them. Consequently, Section 3.2 comprehensively delineates how NETs specifically contribute to metastatic dissemination.

#### 2.3.4. Neutrophils in Cancer Therapy Resistance

Neutrophils contribute to the failure of conventional and targeted cancer treatments by a variety of mechanisms. They protect tumors by creating a network of NETs inside the tumor, creating a physical barrier that prevents the cancer from receiving chemotherapy and immunotherapy [35,61,62]. In addition, they promote immunodeficiency by releasing neutrophil-derived factors that suppress the T-cell response and foster tolerance to immunosuppression [56,63]. Neutrophils also facilitate the repair and growth of tumors by secreting growth factors such as vascular endothelial growth factor (VEGF) and hepatocyte growth factor (HGF), which promote the growth and angiogenesis of tumors [64,65,66]. In addition, neutrophils may aggravate the inflammation associated with treatment and promote a pro-tumorigenic environment, further compromising the therapeutic effect [50,54,67].

#### 2.3.5. Targeting Neutrophils for Cancer Therapy

In view of their multifunctional role, targeting neutrophils has emerged as a promising therapeutic strategy in oncology. One approach is to inhibit the formation of neutrophil extracellular traps (NETs), where deoxyribonuclease (DNase) enzymes can break down these traps and thereby reduce the pro-metastatic effects of neutrophils [28,68].

Another strategy is to block neutrophil recruitment by interfering with the cytokines that attract neutrophils, such as IL-8 and G-CSF, thereby blocking the entry of neutrophils into the tumor microenvironment (TME) [69,70]. Furthermore, the reprogramming of neutrophils by modulating signaling pathways, such as TGF-β inhibition, may shift tumor-related neutrophils towards the anti-N1 phenotype [22,71,72,73]. Combining these strategies with conventional therapies has the potential to improve clinical outcomes and overcome resistance to the treatments [74,75]. A better understanding of the complex interactions between neutrophils and cancer is necessary to design more effective treatments that exploit their potential in cancer immunotherapy [17,76].

## 3. NETs and the Tumor Microenvironment

### 3.1. NETs in Tumor Growth and Progression

NETs can accelerate tumor growth via diverse interrelated mechanisms. The extracellular matrix (ECM) is actively remodeled by NET-associated proteases, such as neutrophil elastase and matrix metalloproteinases, which promotes tumor cell invasion and metastatic spread [14,35,77,78]. Furthermore, depending on the specific receptor and cellular context, NET-associated danger signals trigger pattern-recognition pathways. Specifically, extracellular HMGB1 interacts with the receptor for advanced glycation end products (RAGE) and the TLR4/MD-2 complex, thereby stimulating MyD88-dependent NF-*κ*B and MAPK signaling pathways [79,80]. The activation of these pathways cascades into enhanced tumor-cell survival, endothelial activation, inflammatory cytokine production, and the autocrine/paracrine amplification of NETosis. These mechanisms, however, should not imply that every member of the TLR or RAGE family directly binds to all NET components.

In addition, NETs enhance angiogenesis by releasing MMP9 and VEGF, which stimulate the formation of new blood vessels [30,81,82]. This increased vascularization provides nutrients to the tumor and promotes metastatic spread, which further contributes to the growth and progression of cancer [80].

### 3.2. NET-Mediated Mechanisms of Metastasis

**Figure 2:**
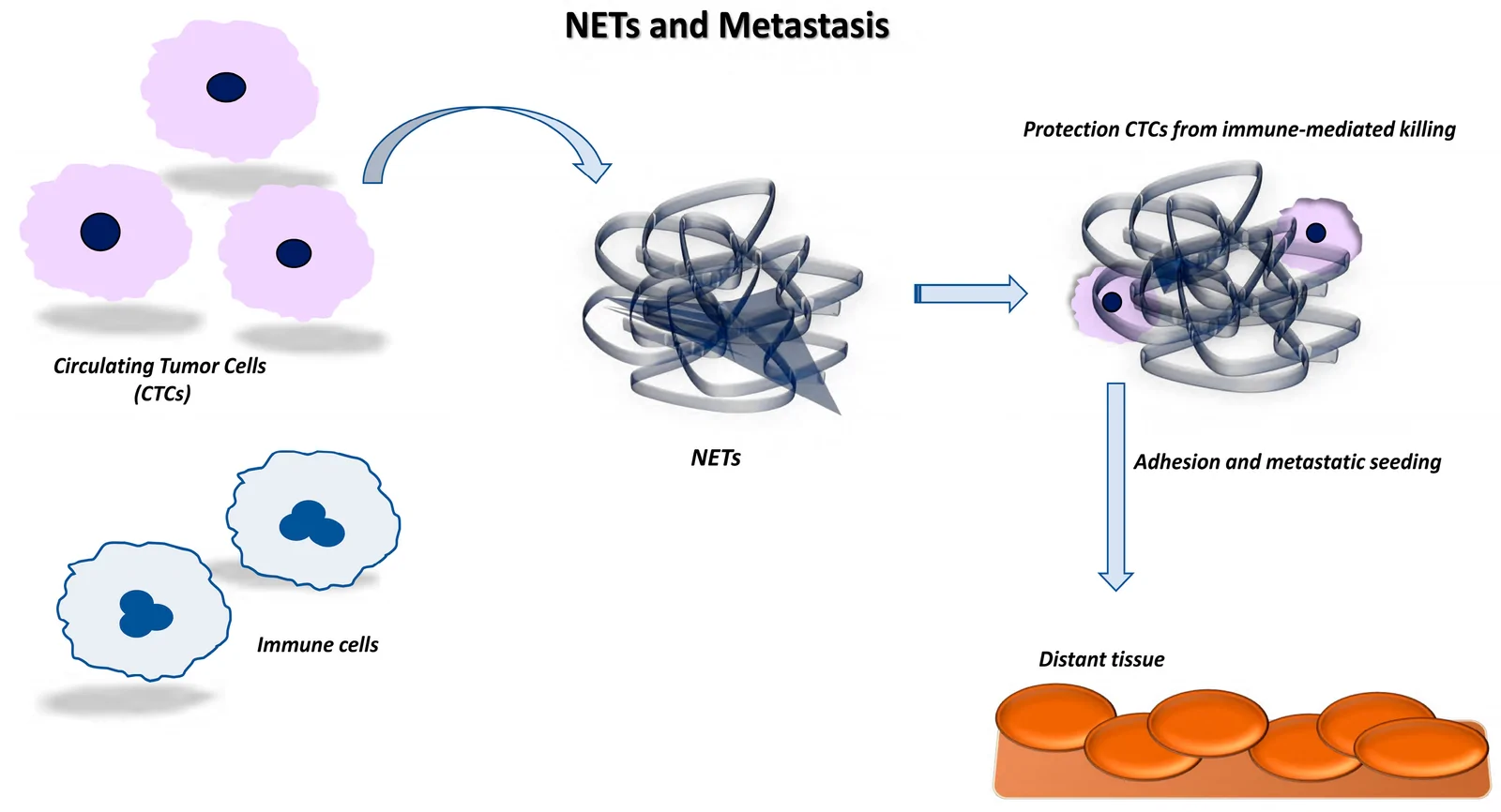
NET-mediated mechanisms of cancer metastasis. Neutrophil extracellular traps (NETs) capture circulating tumor cells and promote their interaction with the endothelium, facilitating vascular adhesion, extravasation, and colonization of distant tissues. NET-associated components may also contribute to immune evasion and the formation of pre-metastatic niches, thereby supporting metastatic dissemination.

NETs can facilitate metastasis through a variety of interrelated mechanisms, although the strength of evidence varies depending on the tumor type and the specific experimental model [35,81]. NETs enhance the adherence of circulating tumor cells (CTCs) to platelets and endothelial cells, prolonging their survival in the bloodstream and aiding in immune evasion by physically ensnaring them [80]. By altering endothelial permeability and interacting with the vascular surface, NETs also promote tumor-cell adhesion and extravasation at distant organs. Additionally, by modulating stromal, endothelial, and immune-cell responses at secondary sites, NET-associated components—such as cell-free DNA, histones, neutrophil elastase, and myeloperoxidase—participate in the formation of pre-metastatic niches [68]. Concurrently, inflammatory mediators and NET-associated proteases drive extracellular matrix remodeling, thereby promoting vascular dissemination and localized invasion [32] (Figure 2).

The relative contribution of NET-dependent versus NET-independent neutrophil mechanisms remains to be fully elucidated, given that the majority of currently available evidence is preclinical. To identify the specific tumor-associated neutrophil states most closely linked to pathogenic NET formation, and to ascertain whether selective NET modulation can restrict metastasis without compromising antimicrobial host defense or antitumor immunity, further research is warranted.

### 3.3. NETs and Therapy Resistance

Neutrophils and NETs contribute to resistance to cancer therapies through multiple interrelated pathways, rendering chemotherapy, radiation, and immunotherapy less effective [35]. One of the primary mechanisms is physical barrier formation. NETs are a dense network structure made up of chromatin, histones, and enzymes produced by neutrophils that surround cancer cells. These physical barriers prevent treatments such as chemotherapy from reaching the core of the cancer. As a result, the concentration of cytotoxic compounds in the tumor cells is reduced, thereby diminishing their ability to induce cell death and allowing the tumor to persist in the body [42,43,50,83]. This barrier effect is particularly problematic in solid tumors, where drug administration is already challenging due to irregular vascularization and high interstitial pressure.

In addition to their physical characteristics, NETs also actively support cancer survival by releasing bioactive molecules that help cancer cells tolerate therapeutic stresses. Proteins related to NETs, including neutrophil elastase (NE), myeloperoxidase (MPO), and various growth factors, such as VEGF and HGF, support the recovery of cancer cells following cytotoxic damage [17,30]. These factors can strengthen the repair mechanisms of cells and encourage the proliferation of cancer cells, making them more resistant to chemotherapy or radiation. Furthermore, NETs contribute to inflammation, which may further maintain a tumor-supporting microenvironment conducive to drug resistance [12,50].

NETs also play a critical role in inhibiting the immune checkpoint and reducing the efficacy of immunotherapies [61] by releasing mediators derived from neutrophils, such as arginase-1 and reactive oxygen species (ROS). These mediators inhibit T-cell activation and proliferation thereby reducing the ability of cytotoxic T-lymphocytes to recognize and kill tumor cells [53,84]. Evidence is growing that NETs contribute to therapeutic resistance in immunotherapy blockade therapies targeting the PD-1 and PD-L1 axis by maintaining an immunosuppressive tumor microenvironment. This inhibition further reduces T-cell recovery and diminishes the therapeutic benefit of checkpoint inhibitors [61].

In summary, NETs contribute to resistance against cancer therapies by creating physical barriers to drug delivery, promoting tumor cell survival by releasing growth factors, and suppressing the immune response, thereby reducing the effectiveness of immune checkpoint inhibitors. Taken together, the multifaceted role of NETs highlights their potential as a therapeutic target to enhance the effectiveness of existing cancer therapies.

### 3.4. Targeting NETs in Cancer Therapy 

Targeting NETs, due to their pro-tumorigenic role, represents a promising therapeutic approach to limit tumor progression and overcome resistance to therapy [12,35]. Several strategies are being studied to disrupt the formation, function, and impact of NETs in the tumor microenvironment [12]. One approach is DNase therapy, which is designed to degrade the DNA backbone of NETs and thereby disrupt their structure. DNase enzymes, such as DNase I, break down the DNA backbone of the NETs and dismantle the physical barrier they form around the tumor. This not only reduces the spread of cancer by limiting the ability of CTCs to spread but also improves drug delivery by increasing the penetration of chemotherapy and immunotherapy into the tumor tissue [12,32]. Preclinical studies suggest that DNase therapy may be particularly effective when used in combination with other therapies, as it may reverse the anti-neoplastic cell protection for tumor cells [61].

Another promising strategy is to inhibit the recruitment of neutrophils into the tumor microenvironment. This can be achieved by targeting key chemokines and growth factors that are involved in the transport of neutrophils, such as interleukin-8 (IL-8) and granulocyte-colony stimulating factor (G-CSF). By blocking these signals, the number of neutrophils at the tumor site is reduced, which decreases the production of NETs and the subsequent pro-metastatic and immunosuppressive effects [4,12,17,85]. Inhibitors of IL-8 are currently being studied in clinical trials and have shown potential to reduce neutrophil-driven tumor progression. A more direct way to target NETs is to block the NETosis pathway, the cellular process that drives the production of these traps.

NETosis is triggered by several signaling cascades, including the activation of NADPH oxidase, which produces the ROS required to form the NETs. NADPH oxidase inhibitors such as diphenyleneiodonium chloride (DPI) may suppress NET production by decreasing ROS levels [86,87]. In addition, Peptidyl Arginine Deiminase 4 (PAD4) is a critical enzyme that facilitates the breakdown of chromatin during NETosis. PAD4 inhibitors have shown promising results in preclinical models by inhibiting the formation of NETs and reducing metastasis [86,88].

**Figure 3:**
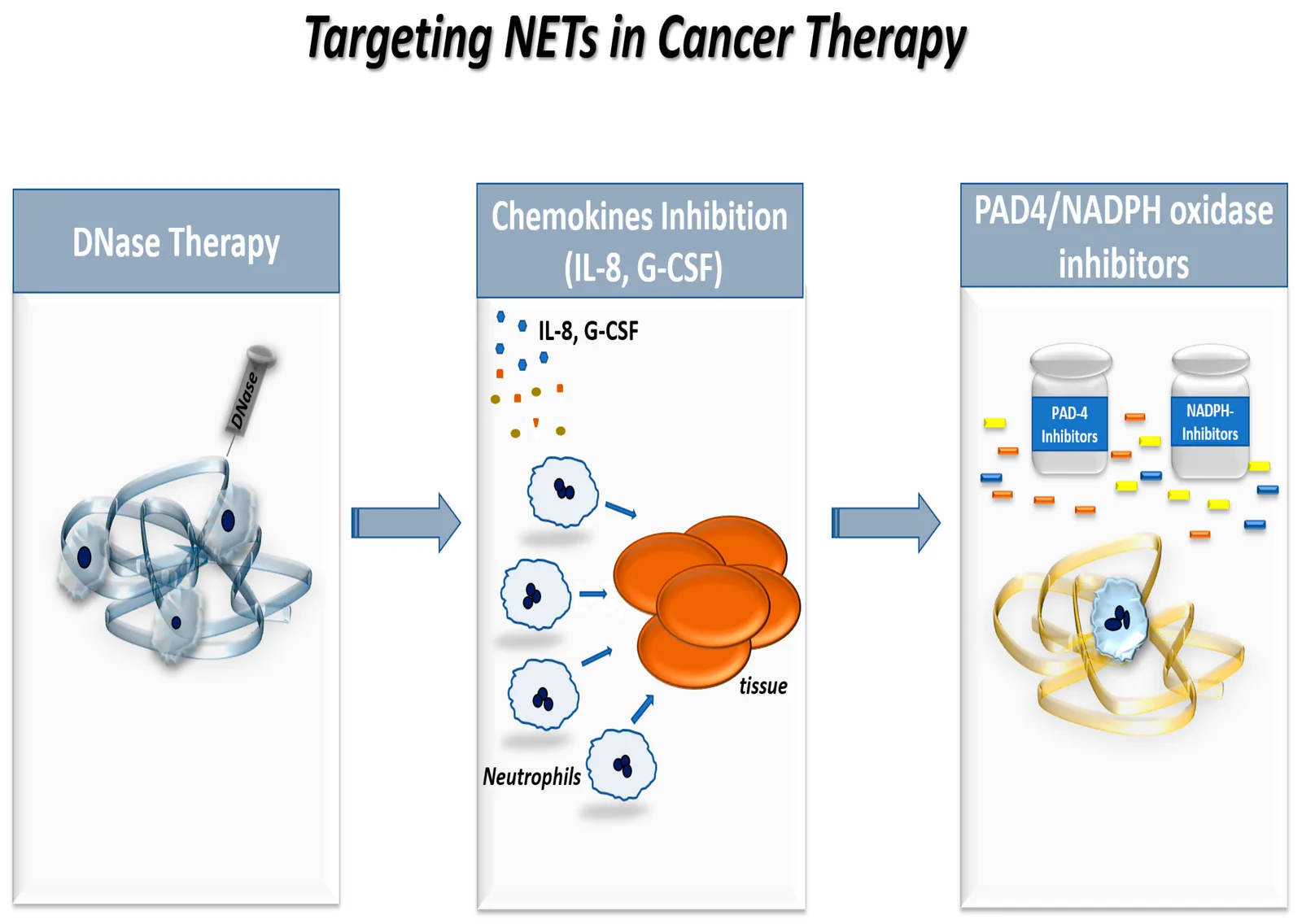
Therapeutic strategies targeting NETs in cancer. Approaches include DNase-mediated NET degradation, inhibition of NETosis pathways (such as PAD4 and NADPH oxidase), and blockade of neutrophil-recruiting chemokines such as IL-8 and G-CSF to limit tumor progression and therapy resistance.

Targeting these molecular pathways may provide a precise and effective means of slowing the progression of cancer driven by NETs. Overall, the focus on NETs represents an emerging and multidisciplinary therapeutic area in oncology. By disrupting the formation and function of NETs, these strategies have the potential to reduce the progression of tumors, improve the effectiveness of existing treatments, and ultimately enhance outcomes for patients. Further research and clinical studies are essential to refine these approaches and translate them into effective cancer therapies (Figure 3).

## 4. Crosstalk Between NETs and Other Cells in the Tumor Microenvironment

### 4.1. NETs and Cancer Cells

It is known that NETs interact with cancer cells in ways that may enhance the cancer progression. NETs have been shown to contribute to the metastatic spread of tumor cells. The physical structure of NETs provides scaffolding to which tumor cells can adhere, helping to prolong their survival in the bloodstream [32,35]. Cancer cells that encounter NETs are more likely to be trapped and then colonized in distant organs, which may increase the risk of metastasis. This has been observed in several tumor types, such as pancreatic, breast, and lung carcinomas [28]. Tumor cells release proinflammatory cytokines such as TNF-α, which can induce the production of NETs and create a positive feedback loop that favors further metastasis [89]. In addition to facilitating metastasis, the components of the NETs system, particularly histones and proteases, interact with tumor cells and stimulate proliferative signaling pathways. Histone inhibitors, which have anti-inflammatory properties, can trigger a cascade of signaling events that promote cell survival and proliferation in tumor cells [15]. Furthermore, NETs contain proteases such as NE, which break down extracellular matrix components, allowing tumor cells to invade the surrounding tissues [17,90].

### 4.2. NETs and Immune Cells

Interactions between NETs and immune cells are complex and bidirectional, affecting both the immune system’s ability to fight disease and its role in supporting tumors. NETs may activate macrophages and monocytes and affect their polarization, leading to a tumor microenvironment (TME)-induced immunosuppressive environment. Specifically, NETs induce cytokines such as interleukin-1β (IL-1β), interleukin-6 (IL-6), and TNF-α to recruit other immune cells, which may favor tumor growth [13,78]. For instance, NETs may induce the polarization of macrophages to the M2 phenotype, which is generally associated with tumor progression, immune suppression, and angiogenesis [91].

Another critical dimension of the TME is the complex interaction between NETs and T cells. Although T cells are key orchestrators of adaptive immunity, their functional capacity is profoundly modulated by NETs [12,26]. By creating a physical barrier that restricts CD8+ T-cell infiltration and direct contact with tumor cells, NETs actively hinder antitumor immune responses [61], while NET-associated signals suppress T-cell activation and impair their cytotoxic capacity. Consequently, an elevated NET burden correlates with diminished CD8+ T-cell infiltration and sub-optimal responses to immune checkpoint blockade across diverse oncological contexts [92]. Whether NETs directly drive immunotherapy resistance or merely reflect a broader, pre-existing immunosuppressive tumor microenvironment remains to be fully elucidated.

In some cancers, such as melanoma, NETs have been shown to reduce the number of functional T-lymphocytes in the TME and enhance the tumor’s immune-evasive capacity [93,94]. NETs also affect dendritic cells, which are involved in presenting antigens and triggering an adaptive immune response. NETs may alter the function of dendritic cells by interfering with their ability to recognize and bind to antigens, reducing the effectiveness of the adaptive immune response to tumors [28,93].

### 4.3. NETs and Endothelial Cells

The endothelial cells that make up the blood vessel lining also interact with NETs in the tumor microenvironment (TME). NETs may modify the phenotype of endothelial cells, thereby promoting angiogenesis (the formation of new blood vessels) and increasing the permeability of the blood vessels [30,95]. By enhancing nutrient supply and facilitating invasion, NETs promote tumor growth. They release pro-angiogenic factors such as VEGF, driving endothelial cell migration and angiogenic vessel formation [96], which supports neovascularization and tumor expansion [30]. NETs may also increase the permeability of endothelial cells, facilitating the extravasation of tumor cells from the bloodstream to distant organs, a key step in the metastatic process [32,97,98].

By directly damaging endothelial cells and disrupting vascular homeostasis during cancer treatment, neutrophils play a key role in cardio-oncology. Chemotherapeutic agents, particularly tyrosine kinase inhibitors (TKIs) and anthracyclines, induce NET formation, which in turn increases vascular permeability, induces endothelial cell death and promotes thrombotic conditions [99,100,101]. When NETs interact with endothelial cells, inflammatory cytokines are released, adhesion molecules are increased, and nitric oxide signaling is impaired. All of these events contribute to endothelial dysfunction and the subsequent cardiovascular complications. The insight into the NET-endothelial axis provides new therapeutic options for protecting vascular integrity and reducing cardiotoxicity in patients with cancer [18].

### 4.4. NETs and Fibroblasts

Fibroblasts are key components of the stroma of tumors, and interactions between fibroblasts and NETs may increase the tumor progression. The presence of NETs may alter the behavior of fibroblasts, resulting in the secretion of pro-tumorigenic factors and extracellular matrix (ECM) remodeling [102]. NETs may activate fibroblasts and stimulate the secretion of growth factors, such as TGF-β, which in turn causes the fibroblasts to differentiate into cancer-related fibroblasts [13,98]. These activated fibroblasts produce ECM components which facilitate the invasion and metastasis of tumors. Additionally, NETs contain enzymes such as elastase and myeloperoxidase, which can degrade components of the ECM. This degradation promotes the proliferation of both fibroblasts and tumor cells [13,103].

## 5. NETs as Therapeutic Targets in Cancer

NETs are implicated in multiple aspects of cancer biology, including tumor progression, metastasis, and resistance to treatment [13,28,35]. Understanding their role may pave the way for novel therapeutic strategies in oncology. In certain contexts, NETs have been shown to inhibit tumor growth. This is mainly due to their role in trapping CTCs in the blood, which prevents them from colonizing distant organs. By acting as a physical barrier, NETs may reduce the metastatic potential of tumor cells [28,32]. In preclinical models, NETs were shown to inhibit the spread of tumor cells in conditions with impaired immune function. One study showed that NETs may inhibit the migration and colonization of melanoma cells by trapping them in the structure of the NETs [93,94]. Although a clinical benefit has not yet been demonstrated, these preclinical findings provide a solid rationale for investigating NET modulation as a potential strategy to limit metastasis in specific cancer models.

The findings highlight the context-dependent nature of NET biology in the context of cancer. NETs are typically linked to tumor progression, metastasis, immune evasion, and resistance to treatment, but recent evidence suggests that they can also have antitumor effects under specific circumstances [104,105,106]. The balance between these opposing functions seems to be determined by various factors, including the nature of the cancer, the stage of the disease, the composition of the tumor microenvironment, the immune system, and the exposure to treatment [104,105,106]. The duality holds significant therapeutic implications, suggesting that regulating NET activity selectively and contextually may be more advantageous than ignoring NET inhibition [104,105,106].

NETs are also known to interact with other components of the tumor microenvironment, such as immune cells, fibroblasts, and endothelial cells, and often promote the pro-tumor environment [17,98]. However, by modulating the formation and degradation of NETs, the TME may be modulated in a way that inhibits the tumor progression. This concept is currently being explored, including the use of NETs as a biomarker for early detection and response to treatment, especially in cancers such as pancreatic and lung cancer, where the TME is critical to survival [49,93,107].

Although they have potential therapeutic value, NETs are also involved in increasing the resistance of cancer cells to therapies such as chemotherapy, immunotherapy, and radiation therapy. Initially, NETs may protect cancer cells against cell death induced by chemotherapy. The physical characteristics of NETs provide a protective layer that prevents the penetration of drugs into tumor cells, reducing the efficacy of chemotherapy [50,93,107].

By modulating intracellular survival signaling in tumor cells, NETs may facilitate the evasion of apoptosis pathways. In some types of cancer, NETs can protect cancer cells against the cytotoxic effects of chemotherapy by increasing survival signals and physically blocking the chemotherapeutic agents from fully binding to the tumor cells [93,108]. In addition to chemotherapy, NETs contribute to cancer cell resistance to immunotherapy and radiation. By creating an immunosuppressive environment, NETs can inhibit the activity of cytotoxic T lymphocytes (CTLs) and natural killer (NK) cells [13,84,109]. Furthermore, NETs may reduce the effectiveness of radiation therapy by protecting cancer cells against DNA damage [93,110].

## 6. Clinical Evidence, Biomarkers, and Translational Challenges of NETs in Cancer

### 6.1. Clinical Evidence and Biomarkers

Clinical studies in oncology have primarily evaluated NET-associated biomarkers rather than NET-targeted treatments, reflecting the early translational stage of therapeutic development [111,112]. Non-invasive circulating markers—including cell-free DNA (cfDNA), neutrophil elastase–DNA complexes, myeloperoxidase–DNA complexes, and citrullinated histone H3—have been investigated as potential indicators of disease burden, treatment response, metastasis, thrombosis, and prognosis. Elevated circulating NET-associated markers across various malignancies have been linked to advanced disease, metastatic progression, therapeutic resistance, and decreased survival [113]. For instance, increased circulating NET levels correlate with more advanced stages and poorer outcomes in pancreatic cancer. However, because these studies are typically observational and heterogeneous regarding cancer type, disease stage, treatment exposure, sampling time, assay methodology, and biomarker thresholds, these reported associations should not be interpreted as definitive proof that NETs independently drive disease progression or that NET inhibition would invariably improve survival [114,115].

In a cohort of 46 patients with advanced non-small-cell lung cancer undergoing first-line chemo-immunotherapy, higher pretreatment NET levels were associated with decreased CD8+ T cell infiltration and worse survival. Furthermore, increased proximity of NETs to cancer-associated fibroblasts and CD8+ T cells correlated with negative outcomes [116]. These findings support the hypothesis that NETs possess prognostic significance and contribute to an immunosuppressive tumor microenvironment.

Nevertheless, the observational design and limited sample size restrict causal interpretations and necessitate validation in larger, independent cohorts. Similarly, elevated circulating NET-associated markers have been linked to treatment resistance in patients undergoing chemotherapy for colorectal cancer. While these results are biologically consistent and suggest that NET measurements could help identify patients at higher risk of treatment failure, it remains unclear whether NET biomarkers offer independent prognostic value beyond traditional tumor stage, systemic inflammatory markers, performance status, therapeutic regimens, and established clinical risk scores.

### 6.2. Therapeutic Evidence and Limitations

NET-targeting therapeutic approaches are currently being investigated to mitigate thrombosis, treatment resistance, metastasis, and cardiovascular damage. Potential strategies include neutralizing cytotoxic NET-associated components like histones or neutrophil elastase, degrading extracellular NET structures with DNase I, and inhibiting NET formation via PAD4 or NADPH oxidase-dependent pathways. Preclinical research indicates that DNase I-mediated NET degradation can reduce metastatic spread in animal models of breast cancer, while PAD4 inhibition has demonstrated promise in experimental models of NET-mediated tissue damage and cancer-associated thrombosis. Although these results support further translational research, they do not establish clinical efficacy. Specifically, experimental improvements in cardiac function or reductions in NET levels must not be framed as proven therapeutic benefits in patients unless backed by prospective clinical trials.

Clinically relevant risks can arise from the broad inhibition of NET formation. Because NETs assist in pathogen containment and antimicrobial host defense, their non-selective suppression can increase vulnerability to infection, hinder tissue healing, or obstruct antitumor immune responses. These issues are particularly critical for oncology patients whose immune systems are already compromised due to malignancy, chemotherapy, radiation, corticosteroids, immunomodulatory therapies, or coexisting comorbidities. Furthermore, the biological effects of NETs are highly context-dependent. While excessive or persistent NET formation drives tumor progression, cancer-associated thrombosis, endothelial injury, and treatment-related cardiovascular toxicity, specific NET-associated functions are vital for host defense or anti-cancer immunity. Therefore, rather than causing indiscriminate neutrophil depletion or a complete blockade of NET production, future strategies should focus on selectively and temporally modulating pathogenic NET activity.

### 6.3. Clinical Translation

Currently, NET-targeted treatments are promising but remain in the early research stage. Implementing these approaches into clinical practice will require standardized biomarker assays, validated thresholds, precise patient selection, and the prospective evaluation of cardiovascular and oncological outcomes. Consequently, clinical trials should rigorously track infection rates, wound healing, immunological responses, thrombosis, bleeding, myocardial injury, heart failure, and overall tumor response. A critical outstanding question is whether NET-associated biomarkers can accurately identify the patient cohorts most likely to benefit from NET-directed therapy. Future investigations should utilize external validation cohorts, serial measurements, and multivariable models that account for established cardiovascular and thrombotic risk factors, concurrent infection, systemic inflammation, therapeutic exposure, and specific tumor characteristics. Ultimately, only prospective interventional studies can ascertain whether NET-targeted therapies improve patient outcomes beyond existing standard-of-care anticancer, antithrombotic, and cardioprotective regimens [117]. Currently, therapies targeted by the NETs should be considered promising but investigational rather than clinical interventions.

Beyond their established effects on tumor growth and metastasis, NETs are heavily implicated in the development of cardiovascular complications in oncology patients. These pathological outcomes are driven by interrelated pathways, including endothelial activation, platelet activation, immunothrombosis, microvascular occlusion, and fibrotic remodeling. The following section delineates the specific role of NETs in cancer-associated cardiovascular toxicity.

## 7. Cardiovascular Toxicity in Cancer and Cardio-Oncology: NET-Mediated Mechanisms

### 7.1. Inflammatory and Thromboinflammatory Context

In cardio-oncology, the neutrophil-to-lymphocyte ratio (NLR) has been widely investigated as a prognostic predictor. In cancer survivors, an elevated NLR is associated with increased cardiovascular and all-cause mortality, suggesting that neutrophil-driven inflammation could assist in stratifying cardiovascular risk. However, because it is a non-specific inflammatory marker, the NLR should not be utilized as a direct indicator of NET formation [118].

Similar inflammatory and prothrombotic axes have been identified in Philadelphia chromosome-negative myeloproliferative neoplasms associated with a heightened risk of atrial fibrillation. In this context, the mechanisms linking cardiovascular complications to clonal hematological diseases may involve neutrophil activation, NET formation, NLRP3 inflammasome activation, thrombosis, and atrial remodeling. These findings suggest that NET-targeted therapies may serve as valuable therapeutic options, though further research is required to confirm their clinical utility [83].

Neutrophils are increasingly recognized as a key biological link between cardiovascular disease and cancer. Inflammatory signals associated with cancer facilitate neutrophil recruitment and activation, platelet-neutrophil interactions, endothelial dysfunction, and NET formation. These interconnected processes can simultaneously drive tumor growth, cancer-associated thrombosis, vascular damage, and adverse cardiac remodeling. Therefore, rather than focusing on isolated mechanisms, the significance of neutrophils in cardio-oncology must be evaluated through these intertwined inflammatory and thrombo-inflammatory pathways [119,120].

### 7.2. NET-Mediated Vascular and Myocardial Injury

By acting in concert with the endothelium, platelets, leukocytes, and the coagulation system, NETs actively contribute to tumor-induced cardiovascular damage [121]. Specifically, extracellular DNA, histones, neutrophil elastase, myeloperoxidase, and other NET components activate endothelial cells, enhance tissue factor (TF) expression, stimulate platelet adhesion and aggregation, and accelerate thrombin production [122]. These molecular events significantly elevate the risk of arterial or venous thrombotic complications, immunothrombosis, and microvascular occlusion [20].

Concurrently, NET-associated inflammatory mediators facilitate myocardial injury and adverse cardiac remodeling. Within this microenvironment, NETs, cardiomyocytes, macrophages, endothelial cells, and cardiac fibroblasts interact to induce fibrosis, extracellular matrix deposition, oxidative stress, and sustained inflammatory signaling, ultimately leading to myocardial stiffness, diastolic dysfunction, heart failure, and diverse cardiovascular manifestations in oncology patients [20].

Furthermore, experimental data indicate that NETs can directly trigger cardiomyocyte death. Specifically, HMGB1-associated NETs promote cardiomyocyte ferroptosis in doxorubicin-induced cardiotoxicity by activating TLR4-associated signaling. This discovery provides a precise mechanistic link between myocardial damage and NET formation, though clinical validation is required to determine its applicability in humans [20].

### 7.3. Treatment-Related Cardiotoxicity

Cancer therapies can exacerbate NET-mediated cardiovascular injury by triggering tissue stress, oxidative damage, endothelial activation, and the release of damage-associated molecular patterns (DAMPs). For instance, anthracyclines, particularly doxorubicin, induce inflammatory signaling, mitochondrial injury, and oxidative stress, thereby creating a microenvironment that favors neutrophil activation and NET formation [20].

Subsequently, NET-associated molecules participate in driving endothelial dysfunction, platelet activation, microvascular thrombosis, and myocardial injury. Similarly, radiation therapy activates inflammatory and fibrotic pathways by stimulating the generation of reactive oxygen species (ROS) and the release of DAMPs. Within the myocardium, interactions among NETs, cardiac fibroblasts, and inflammatory mediators enhance myofibroblast differentiation, collagen deposition, and pathological remodeling via TGF-β and IL-1 signaling pathways [20].

Immune checkpoint inhibitors (ICIs) represent another critical clinical consideration. While ICI-related cardiovascular toxicities—such as immune-mediated myocarditis, arrhythmias, vascular events, and heart failure—are multifactorial, neutrophil activation and NET formation significantly contribute to this highly inflammatory and thromboinflammatory milieu. Although current evidence supports a strong correlation, the direct causal contribution of NETs to ICI-related cardiotoxicity remains incompletely established. Therefore, NETs should be conceptualized as a potential amplifier of treatment-induced cardiovascular injury rather than a primary mediator of cardiotoxicity [20]. This distinction is vital, given that a significant portion of the existing evidence remains either preclinical or observational.

### 7.4. Clinical Implications and Therapeutic Targeting

The clinical relevance of NETs in cardio-oncology stems from their potential utility as biomarkers for thrombo-inflammatory activity, therapy-induced toxicities, and cardiovascular risk. Candidate markers, such as cell-free DNA (cfDNA) and MPO-DNA complexes, could complement established risk assessment methods, though their clinical value will ultimately depend on assay standardization and future prospective validation [123]. Therapeutic strategies targeting NET formation, degradation, or neutralization show significant preclinical efficacy in mitigating thrombosis, myocardial injury, and tumor progression. However, clinical translation must proceed with caution, as broad NET inhibition could compromise host antimicrobial defense and antitumor immunity. Consequently, future clinical trials must identify specific patient cohorts most likely to benefit and define optimal therapeutic windows to balance cardiovascular and oncological outcomes safely.

## 8. Clinical Studies on NETs and Cancer

Although research into the therapeutic role of NETs in cancer is still ongoing, several clinical studies have started to reveal their importance. Clinical studies are exploring the use of non-invasive techniques as biomarkers to predict cancer progression and response to treatment [111,112]. Elevated levels of NETs have been detected in the serum of cancer patients, indicating that NETs may be an indicator of metastasis or poor prognosis. These findings have implications for the monitoring of treatment effectiveness and the detection of early cancer recurrence [113]. In pancreatic cancer patients, high levels of circulating NETs correlated with an advanced stage of the disease and poorer survival [114,115]. This has led scientists to consider targeting NETs for improved survival in high-risk patients.

In another study, a significant decrease in CD8+ T cell infiltration and a significantly worse survival were associated with high pretreatment NET levels in 46 patients with advanced non-small-cell lung cancer (NSCLC) receiving first-line chemo-immunotherapy. Negative results were also predicted by NET proximity to CD8+ T cells and CAFs. In NSCLC, NETs have the potential to be a biomarker and therapeutic target, as evidenced by their confirmation as an independent prognostic factor [116].

The presence of NETs in blood samples has been shown to correlate with resistance to treatment in patients receiving chemotherapy for colorectal cancer [112,124]. These findings support the hypothesis that targeting NETs may increase the efficacy of conventional cancer treatments. Emerging therapeutic strategies targeting NETs to prevent metastasis and overcome resistance to treatment are being explored.

Several drugs, such as inhibitors of NET formation and enzymes (e.g., DNases) responsible for NET degradation are currently being studied in preclinical models [81,125]. A preclinical study in breast cancer has shown that DNase I, which breaks down NETs, was effective in reducing metastatic spread in animal models, providing strong evidence of the potential for targeting NETs in clinical settings [81].

NETs have been demonstrated in a number of studies to have prognostic and therapeutic relevance in cancer and cardio-oncology [126]. Regardless of the source of the specimen, a 2025 systematic review and meta-analysis of 15 studies involving 5202 patients revealed that higher NET levels were linked to worse overall survival (HR 1.80) and disease-free survival (HR 2.26). Compared to cell-free DNA, MPO/DNA, citrullinated histone H3, and neutrophil elastase showed a more consistent correlation. The prognostic potential of NET-associated biomarkers is supported by these findings, but their current clinical application is limited by methodological heterogeneity and the observational nature of the available evidence [113].

Increased circulating NET biomarkers (cfDNA-neutrophil elastase complexes, MPO-DNA) in breast cancer and lymphoma patients receiving anthracyclines or immune checkpoint inhibitors are associated with an increased risk of chemotherapy-induced cardiotoxicity, venous thromboembolism, and poor cancer outcomes [127,128,129]. While PAD4 inhibitors have shown promise in preventing NET-mediated thrombosis during cancer therapy, recent preclinical studies have shown that DNase I administration reduces NET levels and improves heart function in patients with cardiovascular complications associated with cancer therapy [127]. These results confirm that NET-targeted therapies are potential cardioprotective interventions and support the quantification of NETs as a biomarker for the stratification of cardiovascular risk in cancer patients [117].

In Table 1 we present NET-associated biomarkers’ diagnostic and prognostic significance in cancer patients is assessed in representative clinical studies. The majority of the evidence that is currently available is observational and needs prospective validation, despite the fact that NET biomarkers have been linked to survival outcomes, treatment response, and disease progression.

## 9. Conclusions and Future Directions

NETs have gained recognition as an important factor influencing tumor progression, metastasis, immune dysregulation, treatment resistance, thrombosis, and cardiovascular injury. However, their effects are not uniformly pathogenic, and the current evidence remains insufficient to classify NETs as universally harmful or readily exploitable therapeutic targets. Their functional consequences are likely to vary according to tumor type, disease stage, tissue compartment, treatment exposure, and the composition, extent, and duration of NET formation.

Several important barriers to clinical translation remain. Most mechanistic evidence derives from preclinical models, whereas clinical studies are largely observational and heterogeneous with respect to cancer type, specimen source, NET-associated biomarkers, assay methodologies, and threshold definitions. Although a meta-analysis of 15 studies involving 5202 patients reported an association between elevated NET levels and poorer overall and disease-free survival, these findings support a prognostic association rather than causality, predictive treatment value, or therapeutic efficacy. Furthermore, commonly used NET-associated biomarkers lack complete specificity, and it remains uncertain whether they provide clinically meaningful information beyond established risk factors for cancer progression, inflammation, thrombosis, and cardiovascular disease.

Future mechanistic studies should integrate single-cell and spatial profiling technologies with direct NET quantification, functional assays, and longitudinal sampling. Such approaches may enable the identification of pathogenic NETs generated by specific tumor-associated neutrophil states and clarify the biological contexts in which these NETs are formed. Particular emphasis should be placed on elucidating the molecular mechanisms linking NETs to CD8+ T-cell dysfunction, immunotherapy resistance, thrombosis, myocardial injury, and fibroblast activation. These investigations should also distinguish NET-dependent effects from broader NET-independent neutrophil functions.

Clinical research should prioritize standardized and validated NET assays, predefined biomarker thresholds, prospective multicenter cohorts, and integration with established clinical risk models. Interventional studies are needed to determine whether NET-associated biomarkers improve risk stratification or treatment selection and whether NET-targeted therapies can improve patient outcomes. Such studies should evaluate not only oncologic efficacy but also infection risk, immune competence, wound healing, thrombosis, bleeding, and cardiovascular safety.

At present, NET-targeted strategies should be regarded as promising but still investigational. The most effective therapeutic approach is unlikely to involve indiscriminate neutrophil depletion or complete NET inhibition. Instead, future interventions should focus on selectively modulating pathogenic NET activity in a temporally regulated and context-dependent manner while preserving antimicrobial defense and antitumor immunity. In cardio-oncology, the ultimate goal is to determine whether such an approach can improve both oncological and cardiovascular outcomes while maintaining essential host defense functions.

## Figures and Tables

**Table 1:** Representative Clinical Studies Evaluating NET-Associated Biomarkers in Cancer.

Reference	Cancer Type	Study Design	NET Marker(s)	Main Findings	Clinical Significance
Jin et al. [113]	Pancreatic ductal adenocarcinoma	Observational clinical study	Tumor-infiltrating NETs	High intratumoral NET levels were associated with poorer postsurgical survival	Supports the prognostic value of NETs in pancreatic cancer
Lv et al. [116]	Advanced NSCLC	Retrospective cohort study (n = 46)	Pretreatment NET levels	Elevated NET levels were associated with reduced CD8+ T-cell infiltration, poorer response to chemo-immunotherapy, and shorter survival	NETs may serve as prognostic biomarkers and potential therapeutic targets
Wang et al. [112]	Colorectal cancer	Clinical observational study	NET-associated biomarkers	NET-associated biomarkers were associated with prognosis and chemotherapy response in colorectal cancer	Potential biomarker for prognostic assessment and treatment-response prediction
Wannberg et al. [126]	Patients with suspected cancer	Prospective biomarker study	H3Cit-DNA	Plasma H3Cit-DNA distinguished patients with cancer from patients with non-malignant inflammatory conditions	Highlights the diagnostic potential of NET-associated biomarkers
Lee et al. [113]	Multiple cancer types	Systematic review and meta-analysis (15 studies, 5202 patients)	cfDNA, MPO-DNA, CitH3, neutrophil elastase, and related NET biomarkers	Elevated NET levels were associated with poorer overall survival (HR 1.80) and disease-free survival (HR 2.26)	Supports the prognostic significance of NET-associated biomarkers, although current evidence remains observational

Abbreviations: NETs, neutrophil extracellular traps; NSCLC, non-small-cell lung cancer; cfDNA, cell-free DNA; MPO-DNA, myeloperoxidase-DNA complexes; CitH3, citrullinated histone H3.

## Data Availability

No new data were created or analyzed in this study. Data sharing is not applicable to this article.

## Abbreviations

ADCC	Antibody-dependent cellular cytotoxicity
AF	Atrial fibrillation
CTCs	Circulating cancer cells
DAMPs	Damage-associated molecular patterns
DPI	Diphenyleneiodonium chloride
ECM	Extracellular matrix
EMT	Epithelial–mesenchymal transition
G-CSFs	Granulocyte-colony stimulating factors
HGF	Hepatocyte growth factor
IL-	Interleukin
MMP	Matrix metalloproteinases
MPNs	Myeloproliferative neoplasms
MPO	Myeloperoxidase
NE	Neutrophil Elastase
NETs	Neutrophil Extracellular Traps
NK	Natural Killer
NO	Nitric Oxide
NSCLC	Non-small-cell lung cancer
NLR	Neutrophil-to-lymphocyte ratio
PAD4	Peptidyl Arginine Deiminase 4
ROS	Reactive oxygen species
TANs	Tumor-associated neutrophils
TKIs	Tyrosine Kinase Inhibitors
TME	Tumor Microenvironment
TLR	Toll-like receptors
TGF-β	Transforming growth factor beta
VEGF	Vascular endothelial growth factor
